# The Mosquito: A Human History of Our Deadliest Predator

**DOI:** 10.3201/eid2610.202806

**Published:** 2020-10

**Authors:** Teah Snyder

**Affiliations:** University of Massachusetts, Amherst, Massachusetts, USA

**Keywords:** mosquito-borne diseases, malaria, history of disease, vector-borne infections, mosquitoes

The Mosquito: A Human History of Our Deadliest Predator (Figure) details the interrelation between mosquitoborne diseases and the progression of pivotal historical events. Winegard incorporates his expertise in military history with a comprehensive review of the evolution of various mosquitoborne diseases, and delivers a captivating account of humans’ incessant battle with the mosquito. Each chapter of this nonfiction account details the dynamic ways in which mosquitoes influence human survival in each major period throughout history.

**Figure:**
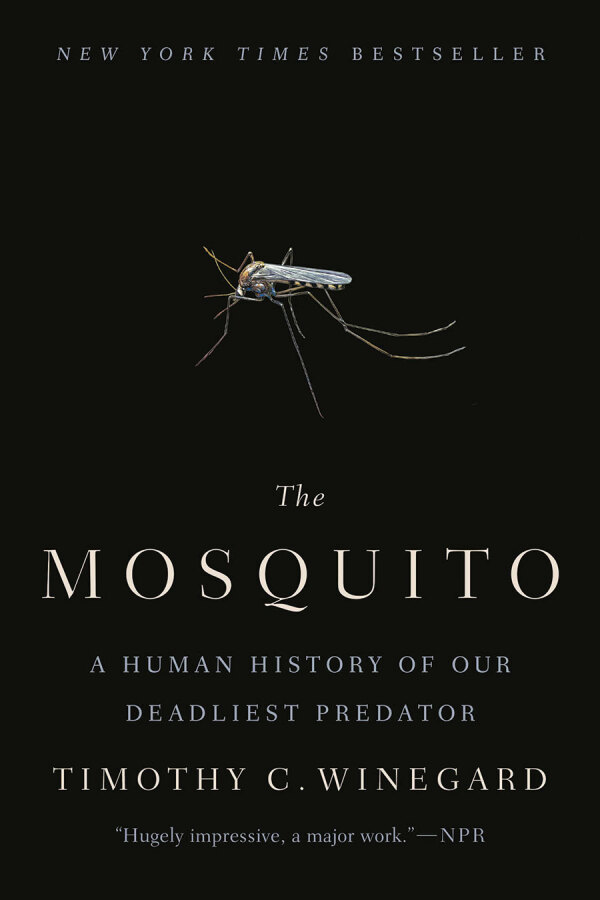
The Mosquito: A Human History of Our Deadliest Predator

This book describes how mosquitoes and their diseases have shaped the outcomes of war, the spread of religion, and the development of modern culture. Attacks from “General *Anopheles*,” which delivered malaria to the Persians as they navigated swampy terrain, ultimately led to a victory by the Greeks during the Greco-Persian Wars. Mosquitoes aided the rise and the fall of the Roman Empire because the Pontine Marshes served as a barrier to enemies and a direct source of disease. Christianity spread across Europe and had a reputation as a healing religion that valued treating persons affected by the mosquitoborne diseases. Christians failed to capture the Holy Land during the Crusades partially because *Plasmodium*-infected mosquitoes attacked inexperienced Crusaders.

Winegard emphasizes the effect of mosquitoborne diseases on the development of the United States. European explorers delivered a lethal dose of mosquitoborne disease to the New World, contributing to the destruction of indigenous populations and the subsequent colonization of the Americas. Partial acquired and genetic immunity to vectorborne diseases drove the demand for enslaved persons from Africa, ensuring the productivity of plantation economies. Widespread malaria delayed the Union victory during the American Civil War, contributing to Abraham Lincoln’s decision to focus on the elimination of slavery. Without malaria, a rapid Confederate defeat might not have led to the Emancipation Proclamation of 1863. Although mosquitoes probably were not the sole reason for these historical outcomes, they most likely contributed substantially to the progression of events.

Winegard emphasizes that, despite modern scientific advancements, the mosquito’s legacy to shape human history is not finished. The development of DDT and antimalarial drugs, such as atabrine and chloroquine during World War II, followed by the subsequent emergence of resistance to these treatments, provide evidence for the need to continue research of mosquitoborne diseases. This book also touches on the controversial topic of clustered regularly interspaced short palindromic repeats, an innovative technology that could genetically alter mosquitoes to prevent human diseases. Although Winegard describes the potential usefulness of this powerful tool, organisms and the environment may suffer unintended devastating consequences.

This book is a fascinating account of the value of mosquitoes in shaping human culture and existence across time. Persons interested in the interplay between history and disease and future implications will learn much and enjoy the accumulation of knowledge and the exciting narrative presentation.

